# Long-Term Visual Outcome and Clinical Predictors Following Yamane Sutureless Intrascleral IOL Fixation

**DOI:** 10.3390/jcm15072523

**Published:** 2026-03-26

**Authors:** Goran Damjanovic, Milenko Stojkovic, Zoran Bukumiric, Mladen Bila, Vesna Sobot, Jana Jaksic

**Affiliations:** 1 Clinic for Eye Diseases, University Clinical Center of Serbia, 11000 Belgrade, Serbia; drmilenkostojkovic@gmail.com (M.S.); mladen.bila@gmail.com (M.B.); janajaksicmfub@gmail.com (J.J.); 2 Faculty of Medicine, University of Belgrade, 11000 Belgrade, Serbia; zoran.bukumiric@med.bg.ac.rs

**Keywords:** Yamane technique, visual outcome, predictor, macular edema, silicone oil removal

## Abstract

**Background**: Sutureless intrascleral intraocular lens (IOL) fixation using the Yamane technique is an option for visual rehabilitation in eyes without capsular support. The aim of this study is to report long-term visual outcomes and clinical predictors in consecutive real-world cohorts, a topic addressed by very few previous studies. **Methods**: This was a single-center, single-surgeon consecutive case series including 87 eyes of 85 patients who underwent Yamane SFIOL for aphakia or lens/posterior chamber IOL ectopia, with at least 12 months of follow-up. BCVA was measured using a Snellen chart and recorded in decimal notation. To identify predictors of postoperative BCVA, univariable screening was first performed, followed by a clinically driven multivariable linear mixed-effect regression. **Results**: Mean age was 68.2 ± 11.4 years, and 70.6% were male. Median follow-up was 26.5 months. Median BCVA improved from 0.2 ± 0.2 (range 0.001–1.0) preoperatively to 0.9 ± 0.2 (range 0.2–1.0) postoperatively (*p* < 0.001). Surgical indication and preoperative comorbidity burden were not linked to postoperative BCVA. In the multivariable analysis, older age (B = −0.005, *p* = 0.027), macular edema (B = −0.242, *p* = 0.035), and prior silicone oil removal (B = −0.237, *p* = 0.046) independently predicted lower postoperative BCVA. **Conclusions**: Yamane SFIOL provides significant long-term visual improvement, with outcomes mainly determined by patient age and retinal status. This study offers new data on functional outcomes and clinically relevant predictors in a consecutive real-world cohort, supporting the reliability and long-term efficacy of sutureless scleral IOL fixation.

## 1. Introduction

Sutureless intrascleral intraocular lens fixation using the double-needle technique, commonly known as the Yamane technique (Yamane SFIOL), was first introduced by Yamane et al. in 2014 [1]. It was later refined with flanged haptics in 2017 [2]. The technique enables secure scleral fixation of a three-piece IOL without sutures. This reduces manipulation and suture-related complications.

Yamane SFIOL is indicated for aphakia, or for dislocation or subluxation of the crystalline lens or posterior chamber IOL in eyes with inadequate capsular or zonular support. Causes may include trauma, complicated cataract surgery, pseudoexfoliation, or prior vitreoretinal procedures. Several modifications of the original technique have been proposed. These address IOL design, haptic configuration, and vitreous management to improve stability and refractive predictability.

Comparative studies of other secondary IOL fixation methods, including Gore-Tex–sutured scleral fixation and Z-suture techniques [3,4], show visual and refractive outcomes comparable to the Yamane technique. A recent systematic review and meta-analysis [5] confirms this. These findings position Yamane SFIOL as a competitive sutureless option. It avoids suture-related complications while maintaining stable functional results.

Several studies describe favorable anatomical and visual outcomes after Yamane SFIOL implantation. However, most focus on surgical feasibility, refractive stability, and complications rather than on clinical predictors of postoperative visual acuity [5,6,7,8]. Available studies often use differing surgical approaches, have limited follow-up, or include trauma-specific patients. This may reduce the broader applicability of their conclusions [5,8]. Data on long-term functional outcomes and independent predictors of postoperative BCVA in real-world, consecutive cohorts remain limited.

This study aimed to evaluate long-term visual outcomes, as measured by best-corrected visual acuity (BCVA), following Yamane SFIOL implantation, and to identify clinical predictors of postoperative BCVA in a consecutive, single-surgeon series with extended follow-up.

## 2. Materials and Method

Study design: This single-center, single-surgeon study included consecutive eyes undergoing Yamane SFIOL for aphakia or lens/posterior chamber intraocular lens (PC IOL) ectopia, with at least 1 year of postoperative follow-up available. Procedures were performed at the Clinic for Eye Diseases, University Clinical Center of Serbia, Belgrade, between January 2020 and December 2024; no Yamane SFIOL procedures were performed in 2021 due to COVID-related restrictions. The study adhered to the Declaration of Helsinki and was approved by the institutional review board of the Medical Faculty, University of Belgrade (approval No. 25/VI-5), and by the Ethics Committee of the Clinical Center of Serbia (approval No. 1600/56), and written informed consent was obtained from all patients. During the preparation of this manuscript, the authors used AI-based language tools (ChatGPT, GPT-5 mini) to assist with improving the clarity and readability of the manuscript. The authors have reviewed and edited the output and take full responsibility for the content of this publication.

Patients and outcomes: All patients underwent a comprehensive ophthalmologic examination. BCVA was measured using a Snellen chart at 6 m and recorded in decimal notation. Eyes were eligible if Yamane SFIOL was indicated for aphakia or lens/PC IOL dislocation with insufficient capsular/zonular support, and if follow-up was at least 1 year. Exclusion criteria included significant keratopathy, corneal scarring, prior corneal transplantation, active uveitis, acute ocular infection, or uncontrolled intraocular pressure (except for urgent cases requiring prompt surgery).

Surgical procedure: Yamane SFIOL implantation was performed according to the previously published technique (2). Anterior or pars plana vitrectomy was undertaken, when indicated, with a 23-gauge Stellaris PC platform (Bausch & Lomb, Rochester, NY, USA) under either peribulbar or sub-Tenon’s anesthesia. Dislocated crystalline lenses were removed via endophacoemulsification or vitrectomy probe, while mature lenses were removed through a 6.0-mm sclerocorneal incision. Dislocated IOLs were explanted through a 3.0-mm limbal incision after optic bisection with endothelial protection. A three-piece MA60AC IOL (Alcon, Fort Worth, TX, USA)) was implanted using the flanged double-needle technique; angled sclerotomies were created 2 mm posterior to the limbus at 3 and 9 o’clock, haptics were externalized through 30-gauge thin-wall needles, flanged with high-temperature cautery, and secured in scleral tunnels. Beginning in 2022, all Yamane SFIOL procedures were performed with a with a custom-designed haptic stabilizer as originally proposed by Yamane.

Statistical analysis: Results were presented as frequencies (percentages), medians (ranges), or means ± standard deviations, depending on variable type and distribution. The Wilcoxon test was used to test statistical hypotheses. Relations between the dependent variable and predictors in repeated-measures designs were examined using generalized linear mixed-effects modeling in R with the lme4 package. Independent variables significant in univariable models (*p* < 0.1) were included in the multivariable model. *p*-values below 0.05 were considered significant. Analyses were performed using IBM SPSS Statistics version 31 (IBM Corporation, Armonk, NY, USA) and R version -4.5.0 (The R Foundation for Statistical Computing, Vienna, Austria).

## 3. Results

Eighty-seven eyes of 85 patients were included. Mean age was 68.2 ± 11.4 years (range, 35–88) and 70.6% of patients were male. Eye laterality was evenly distributed. Surgeries occurred between 2020 and 2024, most often in 2023 (37.9%) and 2024 (40.2%). Baseline characteristics and univariable associations with postoperative BCVA are shown in Table 1.

**Figure 1:**
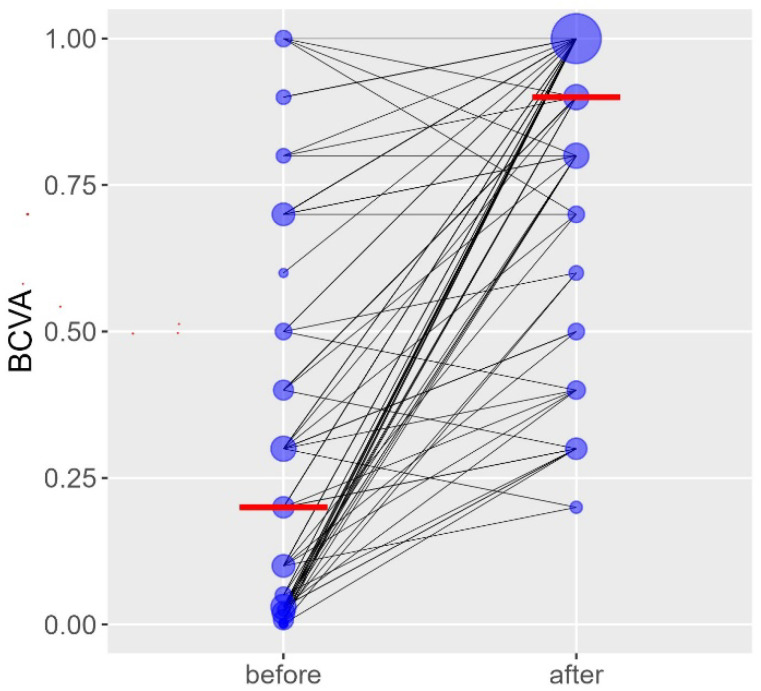
Best Corrected Visual Acuity (BCVA) before and after Yamane SFIOL. Legend: BCVA = Best Corrected Visual Acuity; Before and after refer to Yamane SFIOl. Blue dots: BCVA for individual patients; red lines-group summary (median); grey lines: paired changes.

As shown in Figure 1, median BCVA in Snellen decimal notation improved significantly from 0.2 (range, 0.001–1.0) preoperatively to 0.9 (range, 0.2–1.0) postoperatively (*p* < 0.001). The paired distribution shows a consistent postoperative gain in individual eyes. Most eyes showed a marked increase in visual acuity after surgery.

Median follow-up was 26.5 months (range, 12–68.8). Preoperative spherical equivalent was 5.25 D vs. postoperative 0.02 D (*p* < 0.001). The median IOL power was 21.5 D (range 16.5–26.5 D) and showed no statistically significant association with postoperative best-corrected visual acuity (B = 0.005, *p* = 0.675).Importantly, the primary indication for the Yamane technique, whether aphakia (in 57.5% cases) or lens/IOL ectopia (in 42.5% cases), did not influence postoperative visual outcomes, underscoring the procedure’s comparable functional efficacy across different clinical indications. Similarly, postoperative visual outcomes did not differ by primary indication for the Yamane technique (B = −0.015, *p* = 0.752). Among 37 cases with ectopia, lens subluxation was observed in 9 (10.3%), IOL subluxation in 15 (17.2%), lens luxation in 5 (5.7%), and IOL luxation in 8 (9.2%) cases. Ectopia of the IOL or natural lens was observed in 20 cases (54.1%) with blunt ocular trauma and in 14 cases (37.8%) with pseudoexfoliation. These two factors represent clear causes and/or predisposing conditions for lens/IOL dislocation, including 5 cases (13.5%) presenting with both factors. In the remaining cases, no definitive cause could be identified. By contrast, in aphakic eyes, the underlying causes could be clearly identified. Blunt ocular trauma was present in 20 cases (40%), open-globe injury in 5 (10%), intraoperative IOL implantation failure during phacoemulsification in 21 (42%), and postoperative aphakia in 4 cases (8%).

Preoperative ocular comorbidity showed no significant association with postoperative best-corrected visual acuity, either with respect to its presence (B = −0.074, *p* = 0.182) or the number of preoperative ocular comorbidity (B= −0.028, *p* = 0.281). Table 2 details that blunt ocular trauma, pseudoexfoliation syndrome, capsular glaucoma, and macular edema were significantly associated with postoperative BCVA, unlike other preoperative ocular conditions.

In only 15 cases (17.2%), the Yamane procedure was performed as a standalone surgery, and this did not significantly affect postoperative BCVA (B = 0.022, *p* = 0.072). Additional surgical procedures were commonly performed alongside the Yamane technique, most frequently vitrectomy-related interventions and pupilloplasty. However, none of the additional procedures demonstrated a statistically significant association with postoperative best-corrected visual acuity. Silicone oil evacuation showed a trend toward poorer visual outcomes, although this did not reach statistical significance. The intraoperative use of a Yamane stabilizer, applied in 76 (87.4%) cases, did not show a significant association with postoperative BCVA (B = 0.086, *p* = 0.211). Detailed data are summarized in Table 3.

Postoperative complications were assessed throughout follow-up. No clinically significant issues related to intraocular lens (IOL) stability, such as marked tilt, decentration, or haptic extrusion, were observed. Retinal- and glaucoma-related complications were recorded as part of routine follow-up and managed according to standard clinical practice. As late postoperative complications, retinal detachment occurred in 1 eye (1.1%), developing 20 months postoperatively, and vitreous hemorrhage in 1 eye (1.1%) at 16 months. Clinically significant macular edema was observed in 3 eyes (3.4%), secondary glaucoma in 5 eyes (5.7%), and hypotony in 3 eyes (3.4%).

Ocular trauma was the main reason for sutureless intrascleral IOL fixation, with blunt trauma being more common than open-globe injuries. Open-globe trauma always caused aphakia and did not significantly affect postoperative BCVA (B = 0.085, *p* = 0.393). In comparison, blunt trauma led to varied lens conditions, including aphakia and lens/IOL ectopia. According to univariable analysis (Table 3), blunt ocular trauma was associated with postoperative BCVA; however, this association was only borderline in the multivariable model.

Variables included in the multivariable model were selected based on clinical relevance and univariable screening (*p* < 0.10), prioritizing preoperative ocular status and potential confounders while avoiding collinearity. For example, capsular glaucoma was excluded due to strong collinearity with pseudoexfoliation syndrome and the limited number of cases, which could compromise model stability. In the reduced multivariable model, increasing age was associated with lower postoperative BCVA. Furthermore, as shown in Table 4, macular edema and silicone oil removal remained significant predictors of postoperative BCVA, whereas blunt ocular trauma showed a borderline association, and pseudoexfoliation was not independently significant. Detailed results are presented in Table 4.

## 4. Discussion

Our findings are consistent with contemporary evidence demonstrating that sutureless intrascleral intraocular lens fixation using the Yamane technique provides significant and durable improvement in visual acuity. Similar to large comparative and long-term series, we observed reliable, consistent postoperative BCVA and demonstrated diverse outcomes across different indications [2,6,7].

The magnitude and stability of visual improvement in our cohort align with the seminal description of the flanged thin-wall double-needle technique by Yamane et al. [2] and with subsequent reports across heterogeneous clinical settings, including aphakia, lens/IOL dislocation, and IOL exchange. Importantly, our analysis extends prior work by demonstrating that postoperative visual outcomes are primarily driven by patient- and retinal-related factors rather than by the fixation indication itself.

Ocular trauma was a frequent underlying etiology in our cohort. In agreement with trauma-focused series [8], in this study, blunt trauma was associated with better visual outcomes in univariable analysis; however, this association weakened after multivariable adjustment, suggesting that concomitant retinal pathology, rather than the traumatic mechanism per se, determines postoperative vision. Open-globe injury, although uniformly resulting in aphakia, did not adversely affect postoperative BCVA, further emphasizing the dominant role of retinal status in visual prognosis.

Recent investigations addressing technical nuances of the Yamane technique, including IOL design and intraoperative variations [9,10,11,12,13], consistently demonstrate preserved anatomical stability and refractive accuracy. In line with these reports, we found no significant influence of adjunctive procedures or IOL power on postoperative BCVA. Although not a primary endpoint, accurate IOL power calculation represents an important challenge in secondary IOL implantation. Previous studies report a systematic myopic shift in scleral fixation due to variability in effective lens position (ELP) [14]. In sutureless scleral fixation techniques, such as the Yamane method, the ELP may deviate from standard assumptions, potentially affecting refractive predictability. Variability in haptic fixation and scleral tunnel placement highlights the need for individualized calculation strategies. However, in our cohort, no such trend was observed, and no IOL tilt or decentration occurred. Extended depth-of-focus (EDOF) IOLs may offer a balance between visual quality and spectacle independence; however, their use in scleral fixation remains limited. Enhanced monofocal IOLs provide improved intermediate vision while maintaining a favorable optical profile. Future studies should evaluate their role in Yamane SFIOL fixation. Similarly, vitreous management strategy, anterior vitrectomy versus pars plana vitrectomy, did not affect visual outcomes, corroborating recent comparative analyses [15,16]. Sutureless scleral fixation is a safe and effective option for aphakia or IOL dislocation [17]. Our Yamane three-piece IOL cohort showed stable long-term visual outcomes without tilt, decentration, or major complications, consistent with prior studies comparing scleral flap and pocket techniques [18]. Enhanced monofocal IOLs may improve intermediate vision while maintaining comparable safety [19].

Among preoperative factors, age and macular edema emerged as independent negative predictors of postoperative BCVA. Silicone oil removal was also associated with worse outcomes, likely reflecting underlying vitreoretinal complexity rather than the procedure itself, as previously noted in trauma and vitreoretinal series [8].

Several limitations should be considered. The single-center setting may limit the generalizability of the findings. The wide spectrum of underlying ocular pathology, particularly in trauma-related eyes, may have affected visual outcomes despite multivariable adjustment. In addition, the small number of eyes with certain conditions, such as macular edema or a history of silicone oil removal, may have reduced statistical power. Finally, BCVA was measured using Snellen notation rather than logMAR, which may be less sensitive to subtle changes in visual acuity.

In contrast to prior reports that primarily describe anatomical and refractive outcomes, our study emphasizes clinically relevant predictors of functional visual recovery and demonstrates that postoperative BCVA is driven mainly by retinal status and patient age rather than by surgical indication or technical variables. These findings provide practical prognostic insight for preoperative counseling and case selection in eyes undergoing Yamane SFIOL fixation.

## 5. Conclusions

The strengths of this study include the consecutive case design, a relatively long and uniform follow-up period, and the single-surgeon approach, which reduces procedural variability and facilitates clearer interpretation of clinical predictors. The comprehensive assessment of preoperative ocular status and adjunctive intraoperative procedures, considered as potential predictors of postoperative visual outcome, further strengthens the analysis.

Overall, these findings support the Yamane technique as a reliable and versatile method for secondary intraocular lens fixation across diverse indications. Postoperative visual outcomes are primarily determined by patient age and preexisting retinal pathology, while indication for fixation, traumatic etiology, and additional procedures appear to have limited impact. These findings are important for preoperative counseling and for setting realistic expectations, particularly in older patients and those with macular disease or prior complex vitreoretinal surgery.

## Figures and Tables

**Table 1:** Baseline Demographic and Preoperative Clinical Characteristics of the Study Cohort and Their Association with Postoperative BCVA.

Variable	*n* (%)	B	*p* Value
Age (years)	68.2 ± 11.4 (median 70; range 35–88)	−0.006	0.002 *
Sex (patients)	Male 60 (70.6%); Female 25 (29.4%)	−0.018	0.724
Eye Laterality	Right 42 (48.3%); Left 45 (51.7%)	0.052	0.255
Year of surgery	2020: 4 (4.6%); 2022: 15 (17.2%); 2023: 33 (37.9%); 2024: 35 (40.2%)	−0.010	0.670

Legend: *n* = number; % = percentage; BCVA = best corrected visual acuity; B = univariable regression coefficient; * = statistically significant.

**Table 2:** Pre-Yamane Ocular Characteristics as Predictors of Postoperative BCVA: Univariate Linear Regression Analysis.

Pre-Yamane Findings	*n* (%)	B	*p* Value
Any vitrectomy	53 (60.9%)	0.050	0.282
Pars plana vitrectomy	32 (36.8%)	0.056	0.236
Anterior vitrectomy	21 (24.1%)	−0.032	0.552
Silicone oil present	14 (16.1%)	−0.069	0.270
Blunt ocular trauma	40 (46.0%)	0.141	0.002 *
Pseudoexfoliation	21 (24.1%)	−0.112	0.036 *
Primary open-angle glaucoma	7 (8.0%)	−0.025	0.771
Glaucoma capsulare	8 (9.2%)	−0.278	0.0003 *
Secondary glaucoma	18 (20.7%)	0.077	0.174
Macular edema	3 (3.4%)	−0.250	0.046 *
Epiretinal membrane	8 (9.2%)	−0.051	0.518
Retinal detachment	7 (8%)	−0.070	0.406
ICCE	3 (3.4%)	−0.087	0.492
Endophthalmitis (post)	4 (4.6%)	−0.075	0.495
Hemorrhage (vitreous)	2 (2.3%)	0.130	0.399
Traumatic mydriasis	26 (29.9%)	0.081	0.107

Legend: *n* = number; % = percentage; B = univariable regression coefficient representing the estimated change in BCVA; ICCE = intracapsular cataract extraction; * = statistically significant.

**Table 3:** Additional surgical procedure during Yamane SFIOL as Predictors of Postoperative BCVA: Univariate Linear Regression.

Variable	*n* (%)	B	*p* Value
Any vitrectomy	54 (62.1%)	−0.034	0.478
Vitrectomia anterior via pars plana	17 (19.5%)	−0.060	0.298
Pars plana vitrectomy	23 (26.4%)	−0.022	0.668
Revitrectomia via pars plana	6 (6.9%)	0.119	0.190
Anterior vitrectomy	8 (9.2%)	−0.020	0.798
Silicone oil evacuation	3 (3.4%)	−0.214	0.087
Gas/Air tamponade	12 (13.8%)	−0.001	0.982
Epiretinal membrane peeling	8 (9.2%)	−0.024	0.764
Pupilloplasty	27 (31.0%)	0.061	0.220
IOL explantation	23 (26.4%)	−0.007	0.892
Endophragmentatio lentis	11 (12.6%)	−0.009	0.893
ICCE	4 (4.6%)	−0.066	0.548

Legend: *n* = number; % = percentage; B = univariable regression coefficient representing the estimated change in BCVA; ICCE = intracapsular cataract extraction.

**Table 4:** Variables included in multivariable mixed-effect linear regression model and their influence on postoperative BCVA.

Variable	B	*p* Value
Age (years)	−0.005	0.027 *
Blunt ocular trauma	0.078	0.089
Pseudoexfoliation (PEX)	−0.039	0.476
Macular edema	−0.242	0.035 *
Silicone oil removal	−0.237	0.046 *

Legend: B = regression coefficient representing the estimated change in BCVA; BCVA—Best Corrected Visual Acuity; * = statistically significant.

## Data Availability

The datasets generated and/or analyzed during the current study are not publicly available due to privacy and ethical restrictions involving participant confidentiality, but are available from the corresponding author on reasonable request.

## Abbreviations

The following abbreviations are used in this manuscript:

BCVA	Best Corrected Visual Acuity
Yamane SFIOL	Sutureless intrascleral intraocular lens fixation using the Yamane technique
IOL	Intraocular lens
D	diopter
ICCE	intracapsular cataract extraction
